# Microwave-Assisted Rapid Synthesis of Luminescent Tryptophan-Stabilized Silver Nanoclusters for Ultra-Sensitive Detection of Fe(III), and Their Application in a Test Strip

**DOI:** 10.3390/bios12060425

**Published:** 2022-06-16

**Authors:** Sayed M. Saleh, Wael A. El-Sayed, May A. El-Manawaty, Malek Gassoumi, Reham Ali

**Affiliations:** 1 Department of Chemistry, College of Science, Qassim University, Buraidah 51452, Saudi Arabia; w.shendy@qu.edu.sa; 2 Chemistry Branch, Department of Science and Mathematics, Faculty of Petroleum and Mining Engineering, Suez University, Suez 43721, Egypt; 3 Photochemistry Department, National Research Centre, Dokki, Giza 12622, Egypt; 4 Pharmacognosy Department, Pharmaceutical and Drug Industries Research Institute, National Research Centre, 33 El Buhouth Street, Cairo 12622, Egypt; mayalyem@gmail.com; 5 Department of Physics, College of Science, Qassim University, Buraidah 51452, Saudi Arabia; malek.gassoumi@gmail.com; 6 Laboratory of Condensed Matter and Nanosciences, University of Monastir, Monastir 5000, Tunisia; 7 Chemistry Department, Science College, Suez University, Suez 43518, Egypt

**Keywords:** silver nanoclusters, green synthesis, tryptophan, sensing, Fe(III), anticancer

## Abstract

A new preparation method for extreme fluorescent green emission tryptophan-stabilized silver nanoclusters (Tryp-AgNCs) is presented in this scientific research. The produced silver nanoclusters are dependent on tryptophan amino acid which contributes to normal growth in infants and the sublimation and recovery of human protein, muscles, and enzymes. Herein, we have introduced a green method by using microwave-assisted rapid synthesis. The subsequent silver nanoclusters (AgNCs) have excitation/emission peaks at 408/498 nm and display a considerable selectivity to Fe(III) ions. The tryptophan amino acid molecule was used in the synthesis process as a reducing and stabilizing agent. The Tryp-AgNCs’ properties were investigated in terms of morphology, dispersity, and modification of the synthesized particles using different advanced instruments. The luminescent nanoclusters traced the Fe(III) ions by the luminescence-quenching mechanism of the Tryp-AgNCs luminescence. Therefore, the extreme selectivity of the prepared nanoclusters was exhibited to the Fe(III) ions, permitting the sensitive tracing of ferric ions in the lab and in the real environmental samples. The limit of detection for Fe(III) ions based on Tryp-AgNCs was calculated to be 16.99 nM. The Tryp-AgNCs can be efficiently applied to a paper test strip method. The synthesized nanoclusters were used efficiently to detect the Fe(III) ions in the environmental samples. Moreover, we examined the reactivity of Tryp-AgNCs on various human tumor cell lines. The results show that the Tryp-AgNCs exhibited their activity versus the cancer cells in a dose-dependent routine for the perceived performance versus the greatest-used cancer cell lines.

## 1. Introduction

Iron compounds are distinguished by their numerous applications, including in many industries, such as building materials, drinking water, sewage pipes, food additives, water treatment compounds, dyes in paints, and manufacturing in the plastics’ industry. Various quantities of iron ions are eliminated from the environment and water sources. Iron is not only present in the hemoglobin inside the red blood cells, but it is also used to transport oxygen to vital tissues. Therefore, it is necessary to find advanced or straightforward methods to trace the concentration of iron ions inside cells or in the surrounding environment. In comparison to other transition metal ions, iron is a relatively poisonous metal. The World Health Organization (WHO) set the content limitation of iron ions in drinking water to be 0.3 mg/L [1,2]. Moreover, iron plays a distinctive role in the metabolism and fermentation processes, stimulating the work of various enzymes within the human body. In addition, it acts as a stabilizer for several types of proteins. The uptake of a high quantity of iron or its compounds negatively affects human health and leads to many serious diseases, such as depression, difficulty breathing, nervous convulsions, coma, and heart attack [3,4,5,6]. Most living organisms cannot dispense with iron entirely, but its deficiency or excess can imbalance vital functions. Therefore, scientists have taken seriously the design and development of chemical sensors, to trace the concentration of iron and accurately determine it for use in environmental and health applications [7,8,9,10].

Fluorescent chemosensors are deployed in many applied methods, due to their high sensitivity and rapid response in detecting many different significant ions, including biological molecules [11,12], drug molecules [13,14], pesticides [15,16], or heavy metal ions [17,18,19,20]. They also outperform many devices in shortening the time required to prepare a sample for measurement, such as flame atomic absorption spectrometry [21,22], inductively coupled plasma [23], and inductively coupled plasma mass spectrometry [24], accompanied by many complicated steps that may lead to the loss of the materials to be analyzed. In addition, the analysis-based instrumentation techniques suffer from the low limit of concentration range in which these devices are applied. Additionally, the fluorescent sensors are released to a distinct range up to a picomole. It also facilitates chemosensors usage inside and outside the laboratory, where they are easily carried and applied [25,26,27]. It is worth mentioning that the colorimetric stripe sensors were developed as a simple and inexpensive diagnostic tool. This method is suitable for the naked eye to detect several metal ions in situ. The chemical sensors-based colorimetric strips were reported as a successive tool for detecting metal ions [28,29] and different compounds [30,31] by immobilizing analytical probes. Memorably, different types of fluorescent chemical probes were applied to detect Fe (III) ions. These optical sensors were designed depending on sensing probes such as rhodamine [32], benzofuran [33], glycoluril [34], and modified silver nanoparticles [35].

AgNCs, comprising of accumulation of a limited number of atoms, have a diameter of around 2 nm which is close to the Fermi wavelength. Concerning this considerable small size, the band structures of AgNCs are discontinuous and break down into discrete energy levels, and AgNCs thus give distinctive physical and chemical characteristics. These significant properties involve quantized charging property and tunable fluorescence with high optical stability. The AgNCs are diverse from the larger particle size Ag nanoparticles [36,37]. Typically, AgNCs are produced by reducing the silver ions utilizing reducing and capping agents. Further, AgNCs prefer to accumulate with each other and agglomerate to decrease the internal surface energy. Additionally, the type and chemical structure of the templates such as protein, DNA, and polymers are decisive for the nanoclusters stability [38]. AgNCs were used in different applications such as bio-sensing [39], optical sensing [40], imaging [41], diagnoses of several diseases [42]; and antimicrobial application which is a very promising application. The AgNCs have several advantages compared to the larger Ag nanoparticles with 10 nm, including the ultra-small size with a large surface to volume ratio, the luminescence optical property, and extreme mobility. In addition, these advantages increase the antimicrobial impact of AgNCs, allowing the accomplishment of antimicrobial capacity utilizing a lower concentration of AgNCs than is achievable with Ag nanoparticles [43,44].

**Scheme 1:**
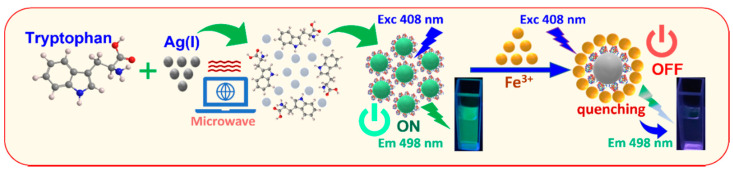
The synthesis scheme of Tryp-AgNCs and their use in Fe(III) ions tracing.

In this work, we used tryptophan amino acid as a momentous chelated ligand as the stabilizing and reducing agents for Tryp-AgNCs synthesis (Scheme 1). The used amino acid has an α-amino group, an α-carboxylic group, and a side chain indole, that initiates this compound to work as a multivalent chelator to reduce the silver ions and stabilize the main metal-core and promote the growth of stabilized luminescent AgNCs. Additionally, we develop for the first time a novel and simple method for the extreme selectivity and sensitivity of Fe(III), utilizing Tryp-AgNCs as a label-free fluorescence strategy. Curiously, it was realized that Fe(III) can quench the luminescence intensity of Tryp-AgNCs. Based on such a result noticed in this research project, an extremely straightforward and fast method for Fe(III) detection was confirmed. Memorably, the Tryp-AgNCs are thought to be an auspicious tool for biological applications.

## 2. Materials and Methods

### 2.1. Materials

The tryptophan, ethylenediaminetetraacetic acid (EDTA), all-metal ions, and chemical compounds were purchased from Sigma Aldrich Co. (Taufkirchen, Germany) (http://www.sigmaaldrich.com, accessed on 20 May 2022). The dialysis membrane of 12–14 KD was obtained from the Spectrum Chemical Co. (New Brunswick, NJ, USA) (https://www.spectrumchemical.com/chemicals/Dialysis-Membrane, accessed on 20 May 2022). All of the used solvents were of the highest grade available.

### 2.2. Apparatus and Instrumentation

The absorbance spectra of the investigated solutions were conducted in a 1 cm quartz cell using an Evolution™ 200 series (Thermo Fisher Scientific, Waltham, MA, USA) UV-Vis spectrophotometer. The luminescence emissions under a certain excitation wavelength were recorded using a JASCOFP8200 spectrofluorometric instrument. The high-resolution transmission electron microscope (HR-TEM) measurements were collected by JEOL-100S Japan, operative at 200 kV. The Fourier Transform Infrared Spectroscopy (FTIR) was used to characterize the synthesized nanoclusters, utilizing a 4100 Jasco-Japan (4000 to 400 cm^−1^). Lastly, the X-ray Photoelectron Spectroscopy (XPS) analyses were applied using a K-ALPHA (Thermo Fisher Scientific, Waltham, MA, USA) with monochromatic X-ray Al K-alpha radiation −10 to 1350 eV spot size 400 µm at pressure 10−9 mbar with full-spectrum pass energy 200 eV and narrow-spectrum 50 eV.

### 2.3. Preparation of Tryp-AgNCs

The Tryp-AgNCs were synthesized in the aqueous medium. Typically, the tryptophan amino acid will be applied in this route as stabilizing and reducing agent. A portion of 4 mL of 10 mM Ag(NO_3_) was mixed with 6 mL of 23.3 mg/mL of tryptophan in water. The prepared mixture was left to stand for three minutes under vigorous stirring, then 0.3 mL 1M NaOH was added to the mixed solution, and the net solution was further stirred for another 3 min. After adjusting the pH to 12.5 using sodium hydroxide solution, the solution was then transferred to a 10 mL microwave sealed tube. After transferring the prepared solution to the microwave tube, the reaction tube was kept in the microwave oven for 20 min (850 W, Anton Paar mono wave 200) with a constant irradiation power. After 20 min, the microwave-assisted Tryp-AgNCs were dialyzed in ultra-pure water for 24 h to remove all of the excess molecules. After dialysis, the Tryp-AgNCs were kept in the dark at 4 °C. The quantum yield of the Tryp-AgNCs was evaluated by using the integrated fluorescence intensities of the nanomaterials and the fluorescein dye that dissolves in ethanol solution as a reference [45].

### 2.4. Detection of Metal Ions

The investigated Tryp-AgNCs’ solution was introduced into an aqueous medium by dilution of the microwave-assisted Tryp-AgNCs 10-fold using a suitable amount of bi-distilled water. The diluted solution was utilized in all of the investigated experiments. After that, the Tryp-AgNCs and 0.1 mM of different metal ions’ stock solutions were prepared, and conducted the luminescence measurements under an excitation of 408 nm. The influence of the various metal cations was examined on the luminescence intensity of Tryp-AgNCs under the same conditions, to detect the sensitivity of the synthesized nanoclusters.

### 2.5. Selectivity and Recovery Measurements

In order to investigate the selectivity and recovery efficiency of the Tryp-AgNCs, another experiment series was conducted by measuring the fluorescence of the as-prepared Tryp-AgNCs in an aqueous solution, after dilution by 10-fold, in the presence of different metal ions and Fe(III) at the same time with an equal molar ratio of metal ion: Fe(III) (1:1). In addition, to study the recovery of the Tryp-AgNCs for four cycles after being applied to the sensing procedure, 20 μM of ethylenediaminetetraacetic acid (EDTA) was mixed with the reacted Tryp-AgNCs and this reaction was repeated after each cycle.

### 2.6. Biological Application—In Vitro Methods

#### 2.6.1. Human Tumor Cell Lines Used

The A431 epidermoid carcinoma, HCT-116 colorectal carcinoma, PC3 prostate adenocarcinoma, A-549 lung adenocarcinoma, MCF-7 breast cancer adenocarcinoma, MIA PaCa2 pancreatic carcinoma, and HOS osteosarcoma cells were kindly supplied by Prof. Dr. Stig Linder, of the Karolinska Institute, Sweden.

#### 2.6.2. Monolayer Cytotoxicity Bioassay

To maintain the cells’ viability, they were sub-cultured twice a week using DMEM-F12 medium, supplemented with l-glutamine and fetal bovine serum added at a concentration of 5–15%, and the flasks were kept at 37 °C incubation under 5% CO_2_ and 95% humidity. The trypsin versing at 0.15 % was used for detaching the cells. When preparing the working 96-well plates, 10,000 to 20,000 cells per well, were seeded, but in the case of the normal cells 60,000 cells per well were prepared, and after a day the cells reached a concentration of about 80%. Under those conditions, the test samples were added in triplicate at 100 µM, 50 µM, 25 µM, and 12.5 µM and the plates were left incubated for 72 h. Positive (Doxorubicin) and negative controls were prepared with the test samples [46,47,48]. MTT [3-(4,5-dimethylthiazol-2-yl)-2,5-diphenyltetrazolium bromide] salt was used to measure cell viability [48]. The resulting blue color was measured at 595 nm with a reference of 690 nm. The percentage viability was calculated by dividing the absorbance of the test well by the negative control and multiplying by 100. If the cytotoxicity percentage is required, we can simply subtract the percentage viability from 100.

#### 2.6.3. IC_50_ Determination

The samples which were shown to be active on the cells were tested. The GraphPad Prism (version 6.01) was used to analyze the results, using the nonlinear regression method, by which we obtained the IC_50_ values.

## 3. Results and Discussion

### 3.1. Tryp-AgNCs Synthesis

The luminescent Tryp-AgNCs were synthesized by mixing tryptophan and AgNO_3_ under certain conditions, as presented in Section 2.3. We noticed that the color of the solution altered to faint brown. Memorably, in the presence of tryptophan, the Ag(I) in silver solution was reduced to Ag(0) based on the indole group [49], where the carboxyl group acted as a protective agent [50]. Thus, tryptophan served as a reducing and protecting agent simultaneously. The procedure needs a template and adjusts the pH. The synthesis procedure was a green, one-step, and straightforward method because all of the utilized reagents were eco-friendly.

### 3.2. Optical Characteristics

**Figure 1:**
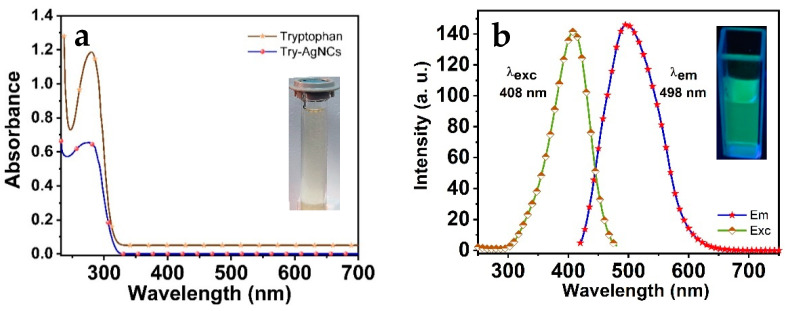
(**a**) The absorption spectra of the tryptophan and Tryp-AgNCs; (**b**) The excitation and emission spectra of Tryp-AgNCs.

Figure 1a exhibits the UV/Vis measurements of the Tryp-AgNCs and the L-tryptophan solutions, respectively. The spectrum of the L-tryptophan solution has a narrow absorption peak. Conversely, the absorption band of the AgNCs has a comparatively broad absorption peak of around 235–330 nm with a peak center of 277 nm. The absorbance of the Tryp-AgNCs was the characterization of the nanoclusters’ size of less than 2 nm and showed their intrinsically molecule-like properties, which is also proof of the presence of the AgNCs. Figure 1b exhibits considerable optical properties, including the emission spectra of the Tryp-AgNCs. The green emission peak of the Tryp-AgNCs was provided at (λ_em_/λ_ex_) of 498 nm/409 nm, respectively. The quantum yield for the as-prepared AgNCs was found to be 37%.

### 3.3. Characterization of Nanoclusters

**Figure 2:**
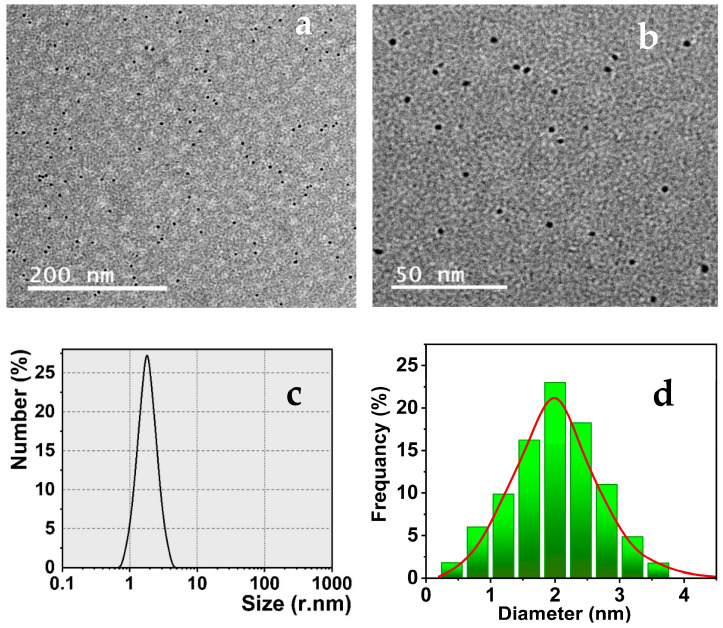
(**a**,**b**) TEM images; (**c**) DLS; and (**d**) SD histogram of the as-prepared Tryp-AgNCs.

To characterize the size and morphology of the synthesized Tryp-AgNCs, the TEM, dynamic light scattering (DLS), and particle size distribution (SD) measurements are presented. Figure 2a,b exhibits the TEM images of the nanoclusters which authenticates that the as-prepared nanoclusters are mono-dispersed and have spherical dots with a uniform diameter of < or ~2 nm, approximately. Thus, the acquired data prove that the green tryptophan reduction process is a substantial method for preparing silver nanoclusters with outstanding performance. The DLS analyses and size distribution (SD) histogram (Figure 2c,d) display that the Tryp-AgNCs have regularly distributed particles and are mono-dispersed in the aqueous solution. The resulting data were agreed with the TEM measurements and the average particle size was estimated to be < or ~2 nm.

**Figure 3:**
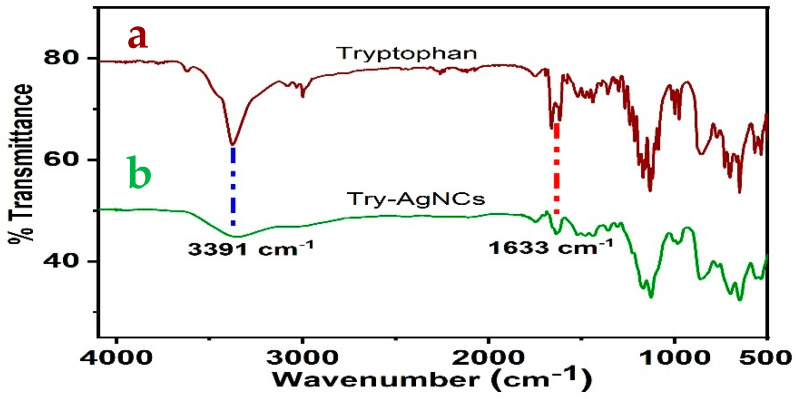
(**a**) FTIR spectra of tryptophan; (**b**) Tryp-AgNCs.

Figure 3 exhibits the infrared spectra of the aqueous solution of tryptophan (a) and the Tryp-AgNCs (b). We can conclude that the distinctive peak of NH_2_ (3025 cm^−1^) [51] in the Tryp-AgNCs was wiped out. Further, the significant peak’s display at 3394 cm^−1^ and 1629 cm^−1^ related to the indole N–H stretching band and the asymmetric stretching band of carboxylate ion, individually. These distinctive peaks confirm the presence of indole 3-acetic acid [52]. The data prove that the oxidation of tryptophan was established into indole3-acetic acid and effectively binds to the AgNCs.

**Figure 4:**
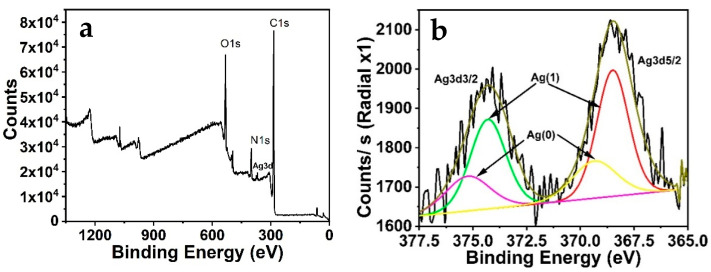
(**a**) XPS spectra of Ag3d for AgNCs; (**b**) The whole XPS spectrum of AgNCs.

To investigate the structure and the composition of the silver nanoclusters, XPS analysis was utilized (Figure 4). The results exhibit two characteristic peaks at 369.28 eV (Ag 3d_5/2_) and 375.18 eV (Ag 3d_3/2_). Further, the binding energy of Ag 3d_5/2_ is in between Ag(0) and Ag(I) (368.46 eV and 374.29 eV), demonstrating the presence of Ag(0) [53]. The peaks were shifted to lower binding energies, showing that the chemical nature surrounding the Ag atoms was altered and this could be attributed to the presence of the mixture Ag(0) and Ag(I). The tryptophan has the structure of an amino acid skeleton, with functional groups including carboxyl and amino groups. So it may reveal that silver atoms interact with N or O of the tryptophan function groups.

### 3.4. Sensing Process

**Figure 5:**
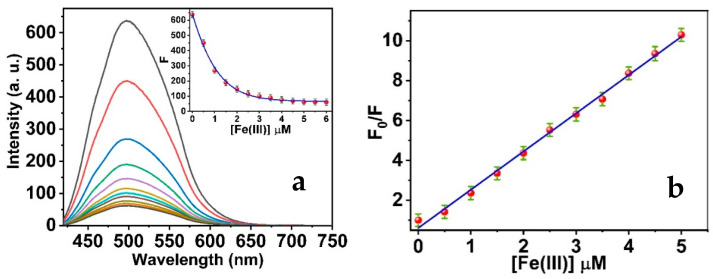
(**a**) Fluorescence emission of Tryp-AgNCs in the presence of a various concentration of Fe(III) ions from 0.1 to 6 mM; (**b**) Classical Stern–Volmer plot of fluorescence intensities ratio versus the quencher concentrations.

Upon the titrimetric reaction of the Tryp-AgNCs in the presence of the Fe(III) solution, the quenching of the green luminescence of Tryp-AgNCs occurred. Figure 5a exhibits the intense green luminescence of the AgNCs that can be clearly quenched by the Fe(III) ions. The consequent emission spectra reveal that the increment of Fe(III) ions diminishes the emission intensity of the Tryp-AgNCs, but does not influence the wavelength of the AgNCs’ maximum peak. In addition, the fluorescence of the Tryp-AgNCs exhibits no spectral overlap with the Fe(III) ions absorption. Thus, the resonance energy transfer cannot be established as the proposed mechanism.

**Figure 6:**
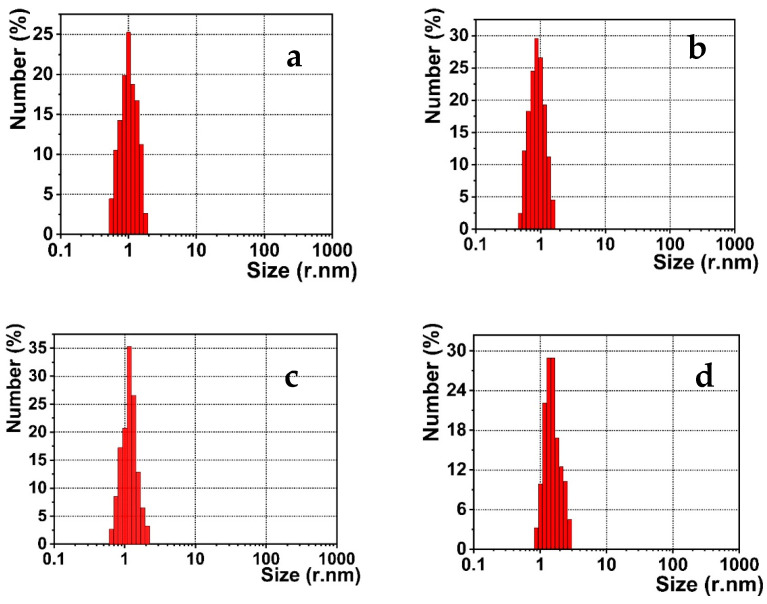
DLS data of Tryp-AgNCs in the presence of (**a**) 0.05; (**b**) 1.5; (**c**) 3.0; and (**d**) 6.0 μM Fe(III).

Our mechanism depends on the hypothesis that the Fe(III) chelated with the AgNCs’ surface via induction of the metal-complex formation with the active functional groups of tryptophan of the Tryp-AgNCs, causing a considerable fluorescence quenching of the Tryp-AgNCs, probably with an electron transfer mechanism. The obtained data suggest that the Tryp-AgNCs exhibit a great selectivity toward the Fe(III) ions. These characteristic properties can be attributed to the significant electron-accepting capability of the Fe(III), and therefore the electrons were simply captured by the Fe(III). The Fe(III) electronic configuration is 3d^5^4s^0^. This increases their charge density and enhances the electron-withdrawing capacity [54]. During the titration process, the Fe(III) ions can be rapidly adsorbed on the Tryp-AgNCs’ surface and establish the complexes through the electron-deficient metal ions and electron-rich tryptophan amino acid [55], escorting to the static fluorescence quenching of the Tryp-AgNCs. The proposed reaction has been proved by a significant association constant between the Fe(III) and Tryp-AgNCs. In the concentration range from 0.05 to 6.0 μM Fe(III), a Stern–Volmer equation, F_0_/F = 1 + 1.9 × 10^6^ M^−1^ [Fe(III)], R^2^ = 0.9956, can be fit, providing a substantial association constant (K_sv_) of 1.9 × 10^6^ M^−1^ (Figure 6b). This proposes an extremely considerable static quenching of the fluorescence emissions of the Tryp-AgNCs by the presence of the Fe(III) ions. Additionally, the limit of detection (LOD) of the studied sensing system was calculated to be 16.99 nM, assuming that the fluorescence emissions can be estimated with a precision of ±1% [56].

Several pieces of research reported the AgNCs that were stabilized with different scaffold molecules. These nanoclusters were utilized to detect the significant metal ions based on the nanoclusters’ aggregation [57,58]. This behavior is generated by the metal-complex formation between the surface scaffold molecules and the effective metal ions. However, this suggested mechanism was inconsistent with our DLS measurements’ resulting data (Figure 6). It is distinct that the size of the Tryp-AgNCs does not alter significantly in the presence of the Fe(III) ions within a series of different concentrations, including 0.05, 1.5, 3.0, and 6.0 μM. No accumulation was detected for the titrimetric samples. Thus, we confirmed that the quenching of emission intensity was not produced by the agglomeration of the Tryp-AgNCs into bigger aggregated particles.

### 3.5. Sensitivity and Recovery

**Figure 7:**
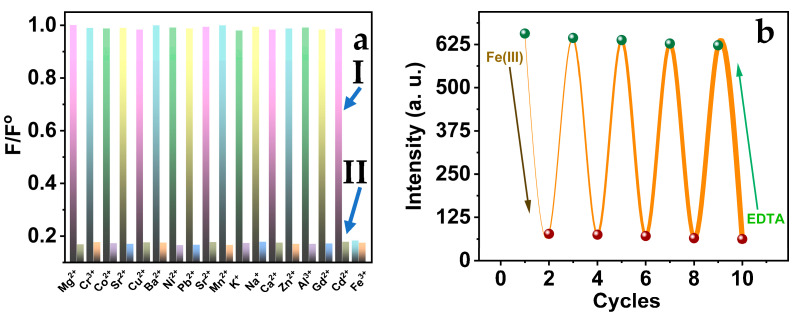
(**a**) Tryp-AgNCs fluorescence intensities in the presence of (I) each different metal ions and (II) each different metal ions and Fe(III); (**b**) The recovery effect of EDTA solution on Tryp-AgNCs fluorescence.

To study the selectivity of the Tryp-AgNCs, the influences of several metal ions on the synthesized nanoclusters were investigated under the same experimental conditions (Figure 7a). No considerable restoration of the fluorescence intensity was observed in the presence of the utilized cations, which proves that the AgNCs can effectively detect Fe(III). In addition, the Fe(III) was used to reduce the fluorescence emission of the AgNCs with electron transfer. The quenching consequence of the Fe(III) can be ascribed to the functional groups of the tryptophan amino acid. Moreover, the EDTA solution greatly enhances the AgNCs fluorescence in the Fe(III)–AgNCs system, because the Fe(III) cations react to the functional groups of EDTA molecules. Therefore, the Fe(III) can be wiped out from the Tryp-AgNCs’ surface successfully, which restored the emission of the AgNCs. Curiously, the recovery of the Tryp-AgNCs fluorescence was attained by 97.8% percent approximately of their initial emission in the presence of EDTA, as shown in Figure 7b. The fluorescence of the AgNCs does not exchange as the number of cycles enhanced and attained a similar fluorescence after reaching four cycles. Moreover, the kinetic alterations in the cycles’ intensities of the AgNCs make for significantly selective Fe(III) ions. Therefore, the recovery of the Tryp-AgNCs method for the Fe(III) detection was acceptable over four cycles.

### 3.6. Test Strips Application

**Scheme 2:**
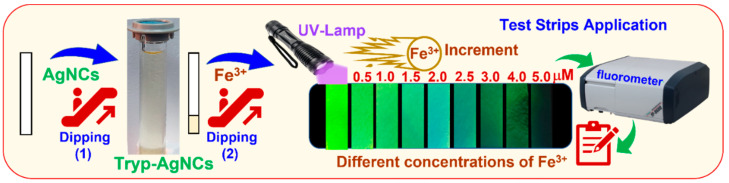
Test strips application-based Tryp-AgNCs for tracing Fe(III).

The Tryp-AgNCs, as a new optical sensor, can be applied to a paper test strip method. The Tryp-AgNCs were spread onto a Whatman^®^ cellulose paper strip with a high-quality cotton liner that is modified to provide an α-cellulose content of more than 98%. The sensing nanoclusters were embedded by the tryptophan scaffolds, which can be entrapped in the test strip. The free unbind Tryp-AgNCs were washed out with bi-distilled water. The test strips’ sensitivity toward the Fe(III) ions was investigated by immersing the strips in different Fe(III) concentrations (0 to 4 μM), as shown in Scheme 2. The test strips also display various intense green luminescence after being immersed in Fe(III) solutions based on the concentration of Fe(III) ions, as shown in Scheme 2. Therefore, the designed strips can be utilized to rapidly detect the Fe(III) concentrations.

### 3.7. Determination of Fe(III) Ions in Real Samples

The tracing of the Fe(III) cations was studied, using the Tryp-AgNCs in real samples in mineral and tap water. The examined samples were utilized straightforwardly without applying any extra treatment. Additionally, the definite Fe(III) cation concentrations in bi-distilled water concentrations were prepared in the environmental samples. In particular, the data are dependent on the fluorescence standard relationship obtained in the lab measurements. The Fe(III) cation concentrations in the mineral and tap water samples were effectively detected. The obtained data are shown in Table 1; the recovery values are 97.60%, 99.35%, and 99.54% for Fe(III) in mineral water and 96.60%, 99.05%, and 99.68% for Fe(II) in tap water. All of the obtained data were matched with the conducted data from ICP-MS, confirming that this detection technique is effective for the determination of Fe(III) in the real samples.

In summary, the developed green optical sensor Tryp-AgNCs displayed a lower LOD than that of some previously published optical sensors exhibited in Table 2, and the World Health Organization limit (0.3 mgL^−1^ ~5.4 µM) for ferric ions in the drinking water.

### 3.8. Cytotoxic Activity

**Figure 8:**
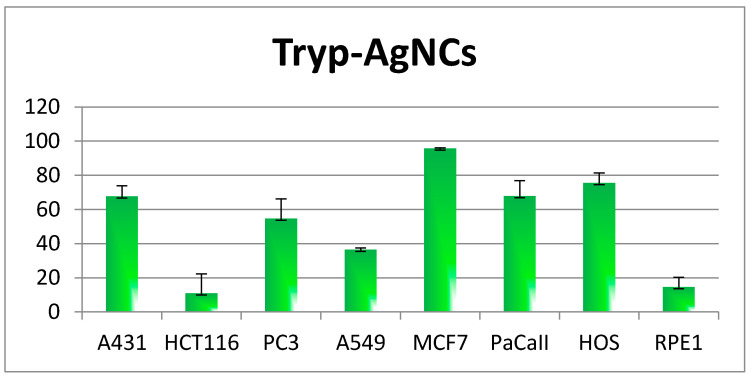
The activity of Tryp-AgNCs on the different tumor cell lines.

The synthesized Tryp-AgNCs compound was investigated for its anticancer activity on a group of human cancer cell lines; human epidermoid carcinoma (A431), colorectal carcinoma (HCT-116), prostate adenocarcinoma (PC3), lung adenocarcinoma (A-549), breast cancer adenocarcinoma (MCF-7), pancreatic carcinoma (PaCa2) and osteosarcoma (HOS) cancer cells. The MTT assay (Mosmann, 1983; Osman ME et al., 2015; Ismail, NS et al., 2016) was applied in the current investigation. The results are shown in Table 3 and Figure 8, displaying the cytotoxic effects of the tested product at 100, 50, 25, and 12.5 µM, whereas Table 4 shows the resulting IC_50_ values which reflected the effect of the synthesized particles on the cancer cells in the current investigation.

It has been revealed that the results of the observed activity, obtained from the tested particle, were generally displayed on the cancer cells in the current investigation, in a dose-dependent manner exhibiting such behavior against the HOS, MCF7, and A431 cancer cell lines. The tested particle, Ag-Tray, exhibited activity from high to moderate against most of the cancers’ cell lines at 100 µM, exhibiting a degree of selectivity at the remaining applied concentrations. The highest activity results were revealed against certain cancer cells, MCF7 and HOS, followed by PC3, A431, and PaCaII. The tested compound showed weak activity against the remaining cancer cell lines, A549 and HCT116. It was found that the MCF7cancer cell was the most susceptible cell at all of the tested concentrations. In addition, the highest inhibition was observed for the MCF7cancer at 100µM, revealing 95.7 ± 0.3 as percentage inhibition. However, although influenced by moderate activity at 100 µM, the PC3 human cancer cell line did not show any observed behavior at the remaining tested concentrations. The same also applies to the PaCaII cancer cells which showed good activity only at 100 µM. Additionally, the most unaffected tested cancer cell at all of the concentrations was HCT116. There is an observed large difference in the displayed activity results on cells such as PC3 and PyCall.

The very small effect observed of the tested particle on the RPE1 Human retinal pigment epithelial normal cell line only at 100 µM, while that cell was not influenced at 50, 25 and 12.5 µM, revealed, obviously, the interesting selectivity of the activity towards the cancer cells with no influence on human normal cells.

## 4. Conclusions

We successfully synthesized the Tryp-AgNCs by using a green, one-step, and straightforward method. The achievement was ascribed to our full knowledge about tryptophan as a reducing and protective agent, making the preparation simple, low-cost, and eco-friendly. The resulting nanoclusters have a green emission under UV excitation. Further, the Tryp-AgNCs effectively provided an optical sensor for detecting Fe^3+^ with a noticeably significant linear relationship between the emission of nanoclusters and the concentration of the Fe^3+^. The activity of the prepared nanoclusters on different human tumor cell lines was studied and the obtained data prove that the Tryp-AgNCs provided their activity against the cancer cells in a dose-dependent way, in terms of the detected behavior against most of the utilized cancer cells lines. Lastly, the results exhibit that the Tryp-AgNCs will be an impactful optical sensor that can detect Fe^3+^ in real samples and biological analysis.

## Figures and Tables

**Table 1:** Tracing of Fe(III) in real samples depending on Tryp-AgNCs (*n* = 3).

Samples	Added Pb(II) µM	ICP-MS µM	Found µM	RSD (%)	Recovery (%)
Mineral water	0.50	0.509	0.488	1.24	97.60
	2.00	2.006	1.987	0.91	99.35
	3.50	3.497	3.484	0.87	99.54
Tap water	0.50	0.517	0.483	1.32	96.60
	2.00	2.009	1.981	0.94	99.05
	3.50	3.505	3.489	0.92	99.68

**Table 2:** Recent optical sensors for ferric ions’ detection.

Synthesis Agent	Type of NPs	Fluorescence Mechanism	Dynamic Range (µM)	LOD (µM)	Refs.
Peptide-6 (GHRP-6)	Au-NCs	Turn-off	2–1000	1.4	[59]
Prunus avium fruit extract	Carbon Dot	Turn-off	0–100	0.96	[60]
Vancomycin	Au-NCs	Turn-off	2–100	1.4	[61]
Fish-scale	Carbon dot	Turn-off	1–78	0.54	[62]
l-proline	Au-NCs	Turn-off	5–2000	2.0	[63]
Boron	Carbon Dot	Turn-off	0−16	242	[64]
l-3,4-dihydroxyphenylalanine	Au-NCs	Turn-off	5–1280	3.5	[65]
Glutathione	Ag-NCs	Turn-off	0.5–2	0.12	[66]
Tryptophan	Ag-NCs	Turn-off	0–6	0.0169	This work

**Table 3:** The activity of Tryp-AgNCs on tumor cell lines.

Cell	100 µM	50 µM	25 µM	12.5 µM
HOS	75.6 ± 5.7	28.2 ± 5.0	22.7 ± 2.2	0.0 ± 0.0
MCF7	95.7 ± 0.3	55.7 ± 3.2	32.9 ± 1.2	5.4 ± 1.0
PC3	54.6 ± 11.5	0.0 ± 0.0	0.0 ± 0.0	0.0 ± 0.0
A549	36.5 ± 0.9	0.0 ± 0.0	0.0 ± 0.0	0.0 ± 0.0
A431	67.7 ± 6.1	3.9 ± 0.5	14.1 ± 0.5	8.2 ± 0.8
HCT116	10.9 ± 11.3	0.0 ± 0.0	0.0 ± 0.0	0.0 ± 0.0
PaCaII	67.9 ± 8.9	0.0 ± 0.0	0.0 ± 0.0	0.0 ± 0.0
RPE1	14.6 ±1.2	0.0 ± 0.0	0.0 ± 0.0	0.0 ± 0.0

**Table 4:** IC_50_ (µM) of Ag-Try on human tumor cell lines.

Cell	IC_50_
MCF7	39.2 ± 5.5, r^2^ = 0.95
HOS	66.3 ± 13.3, r^2^ = 0.89
A431	87.8 ± 1, r^2^ = 0.99

## Data Availability

Not applicable.
